# Carbon Nanofiber versus Graphene‐Based Stretchable Capacitive Touch Sensors for Artificial Electronic Skin

**DOI:** 10.1002/advs.201700587

**Published:** 2017-12-27

**Authors:** Pietro Cataldi, Simeone Dussoni, Luca Ceseracciu, Marco Maggiali, Lorenzo Natale, Giorgio Metta, Athanassia Athanassiou, Ilker S. Bayer

**Affiliations:** ^1^ Smart Materials Istituto Italiano di Tecnologia Via Morego 30 16163 Genova Italy; ^2^ ICub Facility Istituto Italiano di Tecnologia Via Morego 30 16163 Genova Italy; ^3^ Materials Characterization Facility Istituto Italiano di Tecnologia Via Morego 30 16163 Genova Italy

**Keywords:** artificial electronic skin, carbon nanofibers, elongating tactile sensors, flexible electronics, graphene nanoplatelets

## Abstract

Stretchable capacitive devices are instrumental for new‐generation multifunctional haptic technologies particularly suited for soft robotics and electronic skin applications. A majority of elongating soft electronics still rely on silicone for building devices or sensors by multiple‐step replication. In this study, fabrication of a reliable elongating parallel‐plate capacitive touch sensor, using nitrile rubber gloves as templates, is demonstrated. Spray coating both sides of a rubber piece cut out of a glove with a conductive polymer suspension carrying dispersed carbon nanofibers (CnFs) or graphene nanoplatelets (GnPs) is sufficient for making electrodes with low sheet resistance values (≈10 Ω sq^−1^). The electrodes based on CnFs maintain their conductivity up to 100% elongation whereas the GnPs‐based ones form cracks before 60% elongation. However, both electrodes are reliable under elongation levels associated with human joints motility (≈20%). Strikingly, structural damages due to repeated elongation/recovery cycles could be healed through annealing. Haptic sensing characteristics of a stretchable capacitive device by wrapping it around the fingertip of a robotic hand (ICub) are demonstrated. Tactile forces as low as 0.03 N and as high as 5 N can be easily sensed by the device under elongation or over curvilinear surfaces.

## 1 Introduction

Flexible and stretchable (elongating) capacitive devices are indispensable for mechanically compliant advanced biointeractive technologies.^1^ An integrated electronic platform, containing stretchable touch sensors along with other elements such as microscale light‐emitting diodes (LEDs), transistors, resistors, and antennae, that can conform to epidermal topology and equally function in elongated state, may have many implications in biomedical electronics and in humanoid and soft robotics.^2^, ^3^, ^4^, ^5^ Other emerging applications of such devices are unconventional energy storage technologies, such as mechanically deformable supercapacitors,^6^, ^7^ and safety or structural health monitoring in civil engineering.^8^

Inspired by the human tactile performance and the physio‐mechanical architecture of biological tissues and skin, various artificial electronic skin constructs have been fabricated with soft materials to detect several macroscopic stimuli, such as mechanical pressure, stretch, and temperature simultaneously.^5^, ^9^, ^10^, ^11^ Among the most utilized and important elements in artificial electronic skins are the deformable capacitors that can differentiate arbitrary pressure, shear, and torsion induced by a finger.^4^, ^12^, ^13^ Several different flexible and compliant capacitive devices, including pseudocapacitors, have been published to date targeting artificial electronic skin for robotics applications.^14^, ^15^, ^16^ Aside from being flexible, it is equally challenging to ensure that the capacitive devices function under elongation exceeding 100%,^17^ display negligible hysteresis while retaining mechanical robustness against touch and mild friction, are low cost, and can be easily installed on curvilinear topography.^14^, ^16^, ^18^, ^19^ Since fabrication of flexible and strain‐sensitive soft electronics has mainly relied on elastomeric matrices,^20^, ^21^, ^22^ the most utilized approach for the construction of “stretchable capacitors” is to print, deposit, or encapsulate carbon nanotubes (CnTs),^23^ metal nanowires,^24^ liquid metals such as GaInSn or hydrogels^25^ in silicone elastomers^26^, ^27^, ^28^ (polydimethylsiloxane, PDMS) as electrodes. Using elastomeric nanocomposites either as the dielectric layer or as the conducting coating is rather rare, even though they have significant potential for hyperelastic capacitors for soft robots.^29^ Electrofunctional elastomers containing nanoparticles, nanowires, nanotubes, conducting polymers, and quantum dots have been successfully implemented in many sophisticated soft electronic applications.^30^, ^31^ However, as soft robotics technology advances, and many new breakthroughs are reported in epidermal electronic systems,^32^ elongating capacitive devices should be made with much simpler methods by proper design and a careful combination of materials with different mechanical properties.^33^ Moreover, these devices should be “mechanically invisible” in the sense that the elastic properties of the devices must match those of the surface onto which they are applied.^33^

Among nanoscale carbon materials, graphene nanoplatelets (GnPs), multiwalled carbon nanotubes, and carbon nanofibers (CnFs) are already produced in relatively large scale at moderate prices, even though their electrical properties and purity levels are compromised compared to controlled synthesis and purification in laboratory‐scale production.^34^, ^35^, ^36^, ^37^, ^38^ GnPs are in fact made up of many‐layers graphene and are sometimes referred to as graphite‐like nanoflakes.^39^ Due to their thin sheet‐like morphology, GnPs can even be molded or crumpled in situ within the embedding polymer, in such a way that under elongation they can display supercapacitance.^6^ Although very promising flexible capacitors have been developed with graphene or GnPs,^40^, ^41^ novel and simple GnP‐based capacitive devices that can function efficiently under 100% elongation and even more will be very beneficial for soft robotics human interactions. Similarly, buckled carbon nanotubes supercapacitors or strain sensing parallel‐plate capacitors, which function under cyclic stretching, have also been demonstrated.^1^, ^42^, ^43^ In almost all of these capacitive sensors, CnTs were applied or laminated over a silicone elastomer as conductor films (top and bottom electrodes), while the self‐stickiness of the silicone elastomer surface ensured adhesion of the CnTs.^1^, ^42^, ^43^ To the best of our knowledge, CnFs have not been implemented for the construction of elongating capacitive sensors although they were demonstrated as efficient stretchable conductors^44^ and as flexible electrochemical capacitors.^45^

Herein, we demonstrate fabrication of stretchable capacitive devices by a simple spray deposition of rubbery polymer suspensions containing GnPs or CnFs over commercial nitrile rubber glove‐pieces. Therefore, the process is free from complicated multistep fabrication such as mold transfer, etching, templating, or multilayer encapsulation,^46^ and as such can be easily scaled‐up. Since rubber gloves are robust stretchable elastomers, the challenge is to combine them with an equally stretchable and well‐adhering electroconducting coating, in order to sustain capacitance under elongation. For this purpose, we used a polymer blend matrix comprising thermoplastic polyurethane (TPU)^47^ and high impact polystyrene (HIPS),^48^ which demonstrated excellent adhesion to commercial nitrile rubber glove surfaces under tension (>1.0 N m^−1^ with or without GnPs or CnFs). The most conductive elastomeric nanocomposite coatings had ≈10 Ω sq^−1^ sheet resistance, regardless of the type of nanoscale carbon used. As a proof of concept, we fabricated a “plate‐dielectric” capacitive tactile sensor, by spray coating both sides of a nitrile rubber piece, cut out from a laboratory glove, over a 3D‐printed shadow mask that allowed patterning using CnF and GnPs for performance comparison. The capacitive performance was tested under different deformation conditions, in particular by wrapping around a cylindrical object (i.e., fingertip) and under elongation. CnF‐ and GnPs‐based elastomeric capacitors displayed no difference in tactile sensing when they were wrapped around curved objects. On the other hand, however, the CnF‐based elastomeric capacitor was found to be stretch‐stable till 100% elongation, while the GnPs‐based capacitor lost its tactile sensing functionality around 60% elongation. Overall, the fabricated touch sensors are very suitable for facile technologies for human–robot interactions and wearable technologies.

## 2 Results

### 2.1 Conducting Elastomeric Electrode Morphology

**Figure 1:**
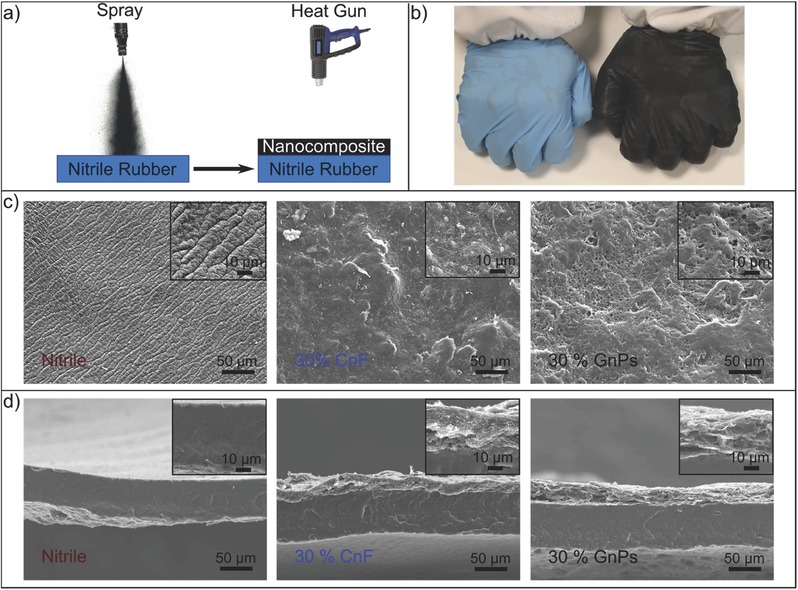
Fabrication and morphology of the nanocomposite coating. a) Fabrication scheme. b) Photographs of a commercial and coated nitrile rubber gloves. c) SEM surface morphology and d) cross‐section SEM images of the bare nitrile rubber, CnF, and GnPs‐based coatings.

Instead of rendering nitrile rubber conductive by incorporating nanostructured carbon during vulcanization,^49^ conducting GnPs or CnFs rubbery inks were sprayed over commercial vulcanized nitrile rubber gloves as seen in **Figure** **1**a,b. Morphology and chemical attributes of both GnPs and CnFs were characterized in detail in our previous reports.^36^, ^37^, ^38^, ^39^, ^44^, ^47^ In short, however, analysis of the Raman spectra indicated that the GnPs are made up of ≈>9 layers^39^ and that CnFs feature many defects/imperfections related to their graphitic structure compared to GnPs (see Figure S1, Supporting Information). Upon a simple heat gun treatment (180–190 °C), the previously sprayed nanocomposites formed a highly conformal coating over the rubber surface, as can be seen in Figure 1b (thermogravimetric characteristics of the conductive coatings are given in Figure S2, Supporting Information). The best performing formulation resulted in a conducting elastomeric coating, which contained 30 wt% GnPs or CnFs presented in Figure 1c. The surface morphology of the uncoated nitrile rubber has ribbon‐like, wrinkled roughness features inherent to its fabrication. These features are known to help coating adhesion.^50^ The surface morphology of both CnF and GnP‐based coatings resembles the morphology of rubber composites made with GnPs or CnFs or CnTs^51^, ^52^ (see also Figure S3, Supporting Information), since the coating matrix is blend of two rubbery polymers, namely, TPU and HIPS (see Figure S4, Supporting Information, for chemical details). The coating/substrate interface is displayed in the cross‐section scanning electron microscopy (SEM) images in Figure 1d. The nitrile rubber thickness is ≈60 µm and the coating thickness is ≈20 µm, as can be seen in these images (Figure 1d). The coatings have very conformal interlocking with the nitrile rubber surface and adapt to its flexibility. Tape peel test results indicate that the adhesion strength (rubber to rubber) was around 1 N m^−1^ and this remained quite stable under elongation particularly for CnF‐based coatings (see Figure S5, Supporting Information). Note that adhesion to steel is a standard performance indicator for coatings or adhesives and adhesion strength of these coatings to metallic surfaces was around 100 N m^−1^.

### 2.2 Electrical Characteristics and Elongation Performance

**Figure 2:**
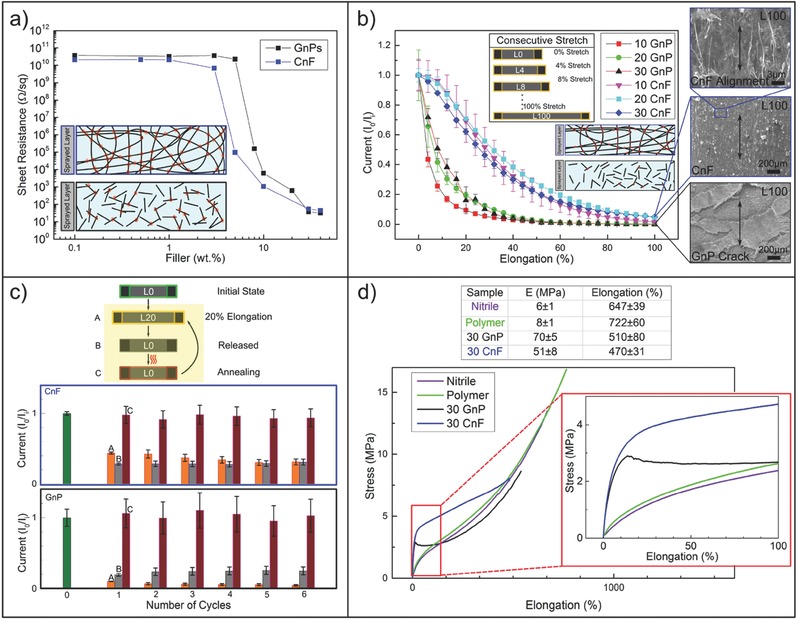
Electrical and mechanical characteristics of the nanocomposites. a) The electrical percolation threshold of CnF and GnPs based samples with a schematic cross section of the sample. b) The stretch test (panel) performed on CnF and GnPs and the current flowing under constant voltage in samples with different nanofillers load (10, 20, and 30 wt%) with the updated elongated schematic cross section. The SEM morphology under 100% stress is also presented. c) The cyclic stretch release cycles scheme performed at 20% elongation and the healing process performed with a simple heat gun process and the relative measurements. d) The mechanical properties of the bare nitrile and of the polymer and their change inserting CnF and GnPs filler.

**Figure** **2**a shows the changes in the coatings' sheet resistance as function of GnPs or CnFs concentrations with respect to the rubbery matrix.^36^, ^53^ Electrical percolation threshold depends very much on the geometry of the nanoscale fillers assuming sufficient dispersion is maintained.^54^, ^55^, ^56^ In general, high aspect ratio wire‐like conductive nanomaterials percolate earlier than nanoparticles, or nanoflakes.^47^, ^54^, ^55^, ^57^ Herein, the length of the CnFs range from 20 to 200 µm, while GnPs' lateral size ranges from hundreds of nanometers to few micrometers^39^, ^44^ (see Figure S3, Supporting Information). Hence, as can be seen in Figure 2, CnFs percolate earlier than GnPs requiring ≈3 wt% less material compared to GnPs. This can be explained by the fact that hundred‐micrometer long fibers can form interconnections easier than few‐micrometer long platelets, as schematically shown in the inset of Figure 2a. Note that the sheet resistance of the CnF‐based coatings corresponding to 10 wt% is nearly an order of magnitude lower than the GnP‐based coatings, while at higher concentrations (i.e., 30 wt%) both nanocomposite coatings reach a saturated sheet resistance value of ≈10 Ω sq^−1^ even under higher loadings like 40 wt%. Above 10 wt% GnPs or CnFs concentration, the nanocomposite coatings demonstrate very stable and hysteresis free ohmic *I*–*V* curves (see Figure S6, Supporting Information, for *I*–*V* measurements).

The different structural geometries of CnFs and GnPs also play an important role for the electrical performance under elongation. We measured the electric current under elongation as reported in Figure 2b for 10, 20, and 30 wt% CnF and GnPs concentrations. Each sample was elongated up to 100% (L0˗L100) with 4% steps. A short pause allowed measurement of current at each increment step under a constant bias of 1.0 V (see inset in Figure 2b). Measured currents in all the GnP‐based coatings decline rapidly. Already at 10% elongation, the initial electric current value dropped by about 80%. After about 50–60% elongation, practically no current could be measured. In the case of CnF‐based coatings, however, only about 5% loss in current was observed at 10% elongation, while an average loss of 60% in current was measured at 40% elongation.

Although about 90% of the initial current is lost at 100% elongation for the CnF‐based coatings (i.e., 20 or 30 wt% CnF) under 1 V bias, this translates into only a tenfold increase in sheet resistance, for instance, from 10 to 100 Ω sq^−1^. For stretchable parallel plate capacitors, designed to detect tactile stimulus, such an increase in resistance does not impede the capacitive sensitivity,^4^, ^16^ as will be shown and discussed next. The CnF‐based coatings are affected less than the GnP‐based coatings by the elongation, due to the previously mentioned density of contacts established between long carbon fibers versus thin platelet carbon. Geometry‐driven contact failure probability in the case of GnP‐based coatings is much higher than the CnF‐based coatings as depicted in the inset of Figure 2b.^57^ Moreover, SEM image analysis results revealed that many large‐scale cracks were formed over the elongated GnP‐based coatings (Figure 2b and Figure S7, Supporting Information) compared to the elongated CnF‐based ones. CnF‐based spray coatings resist elongation‐induced cracks and coating failure and, with strain, the CnFs tend to align parallel to the stress direction, as can be noticed in the SEM images of Figure 2b.^44^ Nevertheless, this alignment process diminishes the number of random interconnections among the nanofibers, lowering the current under constant bias.

Interestingly, we found that stretching‐induced losses in electrical performance can be simply healed by annealing with a heat gun procedure, identical to the fabrication process.^38^ Figure 2c demonstrates this effect in both 30 wt% CnF and GnP‐based coatings. Again, under 1 V bias, the change in current was measured when each coating sample was elongated by 20% (step A) and allowed to relax or released (step B), with six consecutive repetitions (extendible to many more cycles but not shown for brevity). After each stretch–release cycles, heat gun annealing (≈180 °C for 1 min) was performed as schematically shown in Figure 2c (Step C). The rationale behind the 20% stretch–release cycle measurements was the fact that human skin on the joints (elbows, knees, fingers) experience such levels of strain under motion.^58^ In Figure 2c, the dimensionless ratio *I*_0_/*I*_i_ between the initial current and the current during each cycle (steps from A to C), is represented with a columnar graph. As seen, in the case of GnP‐based coatings, the second column, namely, the current recovery (step B) is always higher than the sustained current under 20% strain (step A). For CnF‐based coatings, however, an opposite behavior is observed, since the recovered current is either less or equal to (after the fourth cycle) obtained current under 20% strain. Although, the current obtained by the CnF‐based coatings under elongation is higher than the one obtained by the GnP‐based coatings (i.e., *I*_0_/*I* ≈ 0.4 vs *I*_0_/*I* ≈ 0.1), the GnP‐based coatings recover almost to the same level of *I*_0_/*I*_i_ with the CnF‐based coatings (i.e., *I*_0_/*I* ≈ 0.3 vs *I*_0_/*I* ≈ 0.25). Note that both coatings undergo some permanent deformation after release (see Figure S8, Supporting Information), which is reflected in the value of the recovered current. Better recovery observed in GnP‐based coatings can be attributed to their ability to restructure themselves upon release,^17^, ^47^ and to close the cracks, allowing more flake‐to‐flake contacts (see Figure S9, Supporting Information). Partial alignment of the carbon nanofibers upon elongation disrupts the density of randomly formed carbon–carbon contacts and when released, such a system does not restore its original random morphology, possibly reducing the contact density in the coating. Remarkably, however, for all practical purposes, after a simple convective annealing step, both CnF and GnP‐based coatings fully recover their initial currents.

Stress–strain behavior of the coated nitrile rubber is given in Figure 2d. The results are compared among uncoated nitrile rubber, coated nitrile rubber with no CnFs or GnPs (polymer), and coatings containing 30 wt% CnF or GnPs. The nitrile rubber has an elastic (Young's) modulus (*E*) of 6 MPa, a typical value for elastomeric materials.^59^ The corresponding elongation at break was ≈650%. Polymer matrix‐based coatings maintain similar stress–strain behavior to nitrile rubber with slightly enhanced Young's modulus at 8 MPa and increased elongation at break at 720% (see table in Figure 2d). This could be attributed to the high elastomeric characteristics of the TPU component in the polymer matrix (≈900% elongation at break),^47^ and the good interfacial adhesion between the polymer matrix and nitrile rubber. Instead, the coatings made with 30 wt% CnF and GnPs display rather diverse stress–strain behaviors. For instance, both conductive fillers cause an order of magnitude increase in the elastic modulus, namely, 50 and 70 MPa, respectively. As shown in Figure 2b (also see Figure S7, Supporting Information), GnP‐based coatings demonstrate formation of a significant number of cracks under strain close to 60–80%. This behavior is also reflected in their stress–strain curves. Hence, they experience a sudden drop in stress around 15% and follow the nitrile rubber stress–strain trend until rupture. Nitrile rubber samples coated with the CnF‐based coatings display a plastic like stress–strain behavior. Both CnFs and GnPs‐based samples rupture at around 500% strain.

### 2.3 Device Wrapped Around a Robotic Hand Finger

**Figure 3:**
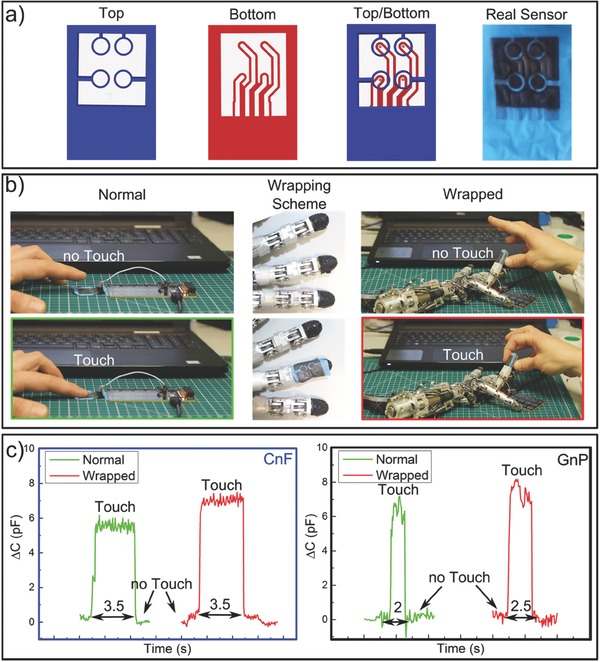
Proof of concept of the tactile sensor functioning in normal conditions and wrapped around a robotic hand finger. a) Scheme of the 3D printed shadow mask employed to pattern the sensor and photo of the top view of the real device; b) setup with the normal and the wrapped configuration; c) touch sensing output from the device unwrapped (green) and wrapped (red) for both CnF and GnPs.

As mentioned earlier, even though conducting coating resistance increases with the elongation of the elastomeric substrate, for stretchable capacitors, this effect is not inhibitive for plate or electrode resistance levels of few kΩs.^4^, ^16^ The elongating capacitive touch sensor was made by using shadow masks fabricated by 3D printing. The mask was designed to pattern conducting pads, lines, and zones for grounding and shielding as well as interlayer connections that enable multilayer circuit tracing. The capacitive design is depicted in **Figure** **3**a. The conductive polymer suspensions were sprayed through the white zones of the design. We designed sensing pads for the top side of the nitrile rubber substrate (blue) and conductive signal paths for the bottom side (red) together with shield regions on both sides for grounding etc. (white zones, see Figure 3a). 300 µm wide holes (see Figure S10, Supporting Information) were laser‐drilled to obtain top‐bottom interconnects (vias) at the required positions. After spraying over the masks with 30 wt% CnF or GnP conducting polymer solutions, the photograph of the final sensor is displayed in Figure 3a (see also Figure S11, Supporting Information).

We tested this flexible and stretchable capacitive sensor by wrapping it around the fingertip of a robotic hand of iCub^60^ with a radius of about 5 mm as shown in Figure 3. Figure 3b shows photographs of the touch sensing experiments conducted on flat and fingertip wrapped devices. The response of both CnF and GnP‐based capacitors is shown in Figure 3c. Upon touch or contact, the tactile sensor produces output signals with comparable amplitude and signal‐to‐noise ratio similar to the flat configuration independent of the conductive coating formulation (Figure 3c). The starting/resting capacitance of the device was of around 5 pF. Upon pressing, for instance, we recorded capacity variations within 6–8 pF, which is designated as ∆*C* in Figure 3c. The presence of a slightly higher signal from the fingertip wrapped device compared to the flat one, can be explained by the reduction in the dielectric thickness due to wrapping‐induced stretching, hence the device has a slightly higher capacitance (*C* ∝ 1/*d* where *C* is the capacity and *d* is the distance between the capacitor plates). The real‐time measurements were also filmed and they can be visualized in Videos S1 and S2 (Supporting Information). Moreover, experiments were repeated with the same devices a month later and using different cylindrical objects with radii ranging from 4 to 20 mm resulting in identical and reproducible responses.

**Figure 4:**
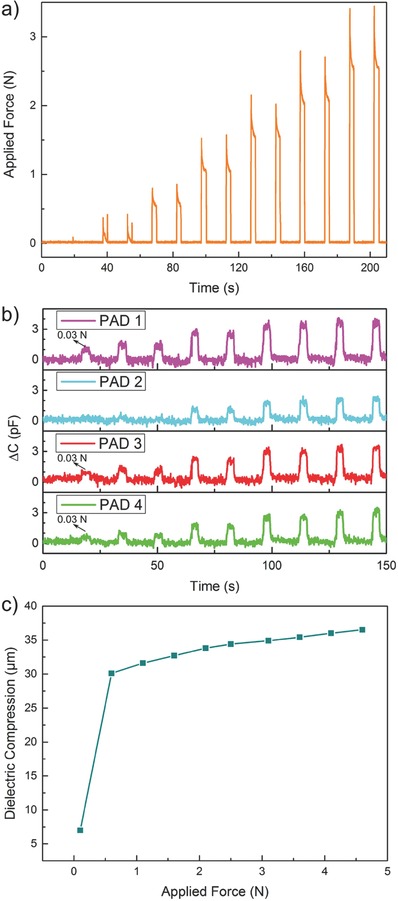
Quantitative analysis of the tactile sensor response. a) Readout of the FT sensor showing force increments of 0.5 N from 0.1 N, two touches for each force value. b) Recorded responses from four taxels. Pink, green, and red were actually pressed by the setup plastic “fingertip” (actual surface ≈ 10 mm^2^) while the cyan one feel “crosstalk” due to the continuity of the dielectric material. Here, the maximum force applied is 2 N. c) Compression of dielectric versus applied force: after a rapid compression at low values of applied force the device shows a linear behavior. This highlights the existence of two concurring elasticity mechanisms and improves response of the device at low contact forces.

In order to gain a more quantitative insight, we used a force torque (FT) sensor mounted on a 3D haptic control. The force was applied by means of a nonconductive fingertip fixed on top of the FT sensor, whose position is finely adjusted by the haptic control. As such, one can apply and measure forces at precise positions on the capacitive device (e.g., on a single or a few sensing pads at once) and correlate them with the capacitance. The response of the FT sensor placed on a single pad or “taxel” is shown in **Figure** **4**a. A gentle touch that is applied twice within 15 s with corresponding force readout of 0.5 N is easily detected. Similarly, the capacitive device functions when larger forces are applied by pressing harder with fingers. Forces exceeding 3 N can be measured in repeatable cycles as seen in Figure 4a. In Figure 4b, capacitance responses obtained from four different taxels are reported. Taxels color coded with pink, green, and red were actually pressed, while the cyan coded untouched taxel transmitted the crosstalk due to the continuity of the dielectric material (nitrile rubber). This is solely based on the design of this capacitive device, and since the human fingertip is larger than the taxel sizes (taxel diameter: 5 mm, fingertip diameter: 10 mm) signals could be detected by adjacent taxels aka force sharing or crosstalk. As seen in Figure 4b, each taxel can detect forces as low as 0.03 N in a repeatable fashion. We also correlated the extent of compression of the nitrile rubber as function of applied force as shown in Figure 4c. The dielectric rubber is compressed by 30 µm under 0.5 N applied force. In other words, from 0.03 to 0.5 N (an order of magnitude increase in force) the compression jumps from 7 to 30 µm (Figure 4c). However, the severity of compression was not as adverse for applied forces between 0.5 and 4.5 N, in fact at 4.5 N applied force the compression was recorded to be ≈37 µm, even though the force increased by an order of magnitude from 0.5 to 4.5 N.

### 2.4 Elongation Performance

**Figure 5:**
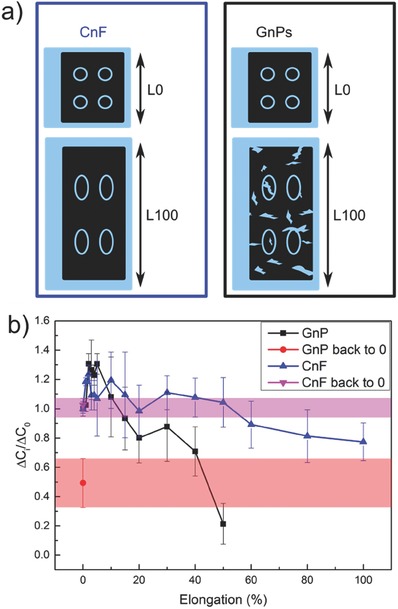
Tactile sensor functionality under stretch. a) Schematic of the CnF and GnPs based tactile sensor mounted at 0% elongation (L0) and at 100% stretch (L100); b) device performances at consecutive elongation steps. ∆*C*_i_ and ∆*C*_0_ represent the capacity variation with touch under elongation and at 0% stretch, respectively. CnF and GnPs based devices behave differently with stretch and release.

Biomechanics of human motion indicates that human skin can elongate between 20 and 40% at the joints.^58^ Our capacitive device, however, was tested up to 100% elongation that may be more realistic for mechanics of extreme soft robotics motion. Devices based on both CnF and GnPs (30 wt%) were stretched in successive steps, similar to the ones used in the electric current versus strain characterization, shown in Figure 2. For each intermediate elongation step, the device was touched with a fingertip with a force ≈0.5 N. Before reaching 100% elongation GnP‐based coatings applied on nitrile rubber suffer from many cracks and discontinuities (see **Figure** **5**a; also see Figure 2b and Figure S12, Supporting Information). CnF‐based coatings maintain their crack‐free uniform texture even at 100% elongation (L100 in Figure 5a). In Figure 5b, we report dimensionless capacitance variation under touch and stretching for both devices. Remarkably, the haptic response of the CnF‐based device under increasing elongation appears to be very stable considering the measured uncertainty levels. On average, only a 20% decrease at 100% elongation is measured. After returning back to the “relaxed” initial state CnF‐based stretchable sensor recovers the initial output. Conversely, the GnP‐based sensor shows a substantial drop in touch response under elongation, with 80% loss in sensitivity at 50% elongation and becoming nonfunctional between 60 and 70% elongation. When returned to initial state after 100% elongation, the sensitivity is reduced by half as shown in Figure 5b. Results in Figure 2b demonstrate that indeed between 60 and 70% elongation, practically no current passes through the GnP‐based coatings. Note that GnP‐based coatings start forming cracks already at 15% elongation (see Figure 2d); whereas as discussed earlier, the sheet resistance of crack‐free CnF‐based coatings at 90% elongation is in the order of hundreds Ω sq^−1^, sufficient for tactile sensing.^4^, ^16^

On the other hand, considering the human biomechanics, that is, skin stretch range between 20 and 40%, on average there is no difference between CnF and GnP‐based coatings in terms of capacitive response under elongation as seen in Figure 5b. Finally, the real‐time stretch responses of the devices are filmed and can be viewed in Video S3 (Supporting Information) for real‐time behavior of GnPs‐based sensor.

## 3 Conclusions

Stretchable capacitors were fabricated by simply spray coating nitrile rubber gloves with conductive rubbery slurries containing carbon nanofibers or graphene nanoplatelets. Conductive coatings applied on both sides of elongating nitrile rubber glove pieces acted as parallel plate electrodes for a soft capacitor device. Sheet resistance of the conducting and conformal coatings was ≈10 Ω sq^−1^. Coatings demonstrated very good adhesion to the nitrile rubber surface and hence withstood different elongation levels depending on GnPs or CnFs. CnF‐based coatings electrically percolated at lower filler concentrations compared with GnPs (3 wt% vs 6 wt%) and resisted elongation without cracks formation. On the other hand, GnP‐based coatings cracked while elongating. Structural damages due to repeated 20% elongation were healed several times by a simple annealing process for both types of coatings.

Both CnF‐ and GnPs‐based elongating capacitive sensors functioned when wrapped around a fingertip of a robotic hand. CnF‐based elongating capacitors functioned well and were stable under 100% elongation while GnP‐based sensors failed. Simplicity of this spray paint process indicates that such rubbery conductors can be painted or printed over any elastomeric surface that is industrially available. The process also avoids use of multistep PDMS methods and clean room machinery.

## 4 Experimental Section

*Materials*: Nitrile rubber (acrylonitrile butadiene) gloves were purchased from NaturSint (high tech gloves). GnPs (grade Ultra G+) were donated by Directa Plus S.p.A (Italy). Graphitized CnF (diameter ≈ 100 nm, length between 20 and 200 µm) was purchased from Sigma‐Aldrich (grade PR‐25‐XT‐HHT from Pyrograf Products Inc.). TPU (Elastollan 1185A) was acquired from BASF and was mixed with HIPS (rubber content ≈8–13 wt%, polystyrene molecular weight 260 000, polybutadiene molecular weight 120 000) as polymer matrix. Typically, the conductive polymeric slurry was prepared by employing 0.5 g of dry polymer blend (75% TPU and 25% HIPS) and a certain percentage of CnFs or GnPs indicated throughout the text as wt% relative to the amount of polymer. For examples, a conductive coating containing 30 wt% GnPs translates into a nanocomposite having 0.15 g GnPs. The solvent employed was chloroform (30 mL every 0.5 g of dry polymer blend) that was obtained from Sigma Aldrich. The conductive slurry was tip sonicated (20 kHz, 750 W, 40% amplitude, 4 times for 30 s) through a Sonics & Materials, Inc. sonicator (Model Num. VCX750) to obtain a homogeneous dispersion. Afterward, 5 mL of dispersion was spray painted (14–16 cm distance, 2.0 bar) on pure nitrile rubber surface (7.5 × 7.5 cm^2^). To ensure solvent evaporation and good adhesion, annealing with a heat gun was employed (≈180–190 °C, 15–18 cm distance, 1 min). This procedure was repeated twice, rotating the samples by 90°.

*Measurements*: SEM images of the surface morphology and the cross section of the samples were acquired with a JEOL microscope (model JSM‐6490LA, acceleration voltage of 15 kV). To cut the specimens for cross‐sectional SEM images, they were frozen in liquid nitrogen and fractured by tweezers.

*I*–*V* curves of various samples for determination of electrical percolation threshold, under stretching and after healing by annealing, were measured with a four‐probe Keithley 2611A sourcemeter. Silver paste (SPI Conductive Silver Paint) was painted creating 5 mm thin contacts on the samples spaced by 5 mm. The effect of stepwise and cyclic deformation on the current of the nanocomposites was characterized by the sourcemeter coupled with a uniaxial testing machine (Instron). The samples were clamped on the testing machine and electrodes were connected to the specimen's ends. 1 V tension was applied and the current without deformation was recorded (*i*_0_). During stepwise tests, the elongation was increased by 4% at each step with a rate of 2 mm min^−1^. At each single step, deformation was kept constant for about 1 min to allow the sample relaxation, and then the current was recorded. For the cyclic tests, each cycle corresponded to an elongation of 20% of the initial length (strain rate of 10 mm min^−1^), then slow released back to zero strain. At the end of cyclic process a simple healing procedure was performed with a heat gun (same parameters as the fabrication process). At each step of the cycle, the current flowing in the samples was measured. The uniaxial testing machine (Instron 3365, strain rate 10 mm min^−1^) was also utilized for stress–strain characteristics of the elongating capacitors. For measurements given in Figure 2, at least five different samples were measured to obtain statistics.

To fabricate the elongating tactile sensor, identical spray coating and heat gun procedure were used over a mask. Before spraying, electric vias was created on the nitrile rubber by laser micromachining. A KrF excimer laser with a 20 ns full‐width at half maximum (Coherent‐CompexPro 110, fluence 2.2 J cm^−2^, 1000 hits) was utilized at 248 nm. The laser was coupled with a micromachining workstation (Optec‐MicroMaster). The nitrile rubber was irradiated using the projection mask technique (square hole shaped, 300 µm lateral side) with a 0.1 numerical aperture projection lens set at a demagnification of 6 with optical resolution of 1.5 µm. Acrylonitrile butadiene styrene (ABS) polymer based shadow masks were designed and 3D printed. Two different designs for the top and for the bottom of the tactile sensor were implemented to create pads and paths, respectively. The pads were touched deforming the nitrile rubber (dielectric of the capacitive sensor). The signals were transmitted to software through the paths in the bottom section. Elongating capacitors were connected to a Silicon Labs CPT112S capacitive sensing IC, having the following features: gain control, noise reduction tools, a dedicated standalone card for rapid prototyping and testing, and an easy to use interface running on PC. Elongating tactile devices made with the 30 wt% CnF or GnPs conductive slurries were tested under no elongation of flexing, wrapped around different cylindrical objects (radii of curvature from few till 0.4 cm), and under elongation (from 0 to 100% of the initial length). The touch tests under elongation were accomplished employing the uniaxial testing machine used for mechanical tests. A minimum of three different haptic devices were tested for each nanofiller type. Touch sensing tests were repeated on multiple pads of each device and averaged. To perform, a quantitative analysis on the tactile sensors, a force torque (FT) sensor ATI Nano‐17 mounted on 3D haptic control omega.3 from Force Dimension was used. Before the quantitative tests, the device was covered with a polyurethane polymeric insulator the top portion of which was spray painted with a layer of conductive 30 wt% GnPs or CnFs nanocomposite to act as a grounded electrode for detection of nonconductive objects. The final polyurethane layer was simply laminated on top of the tactile sensor.

Raman spectra of the carbon‐based nanofillers were acquired with a Horiba HR800UV, LabRAM 600 spectrometer (diffraction grating of 600 line mm^−1^, excitation wavelength of 632.8 nm HeNe laser, maximum power 20 mW). FTIR spectra of the materials (from 600 to 4000 cm^−1^, 2 cm^−1^ resolution, averaging 128 scans) were recorded with a Bruker Vertex 70v. Thermogravimetric analysis of the nanocomposites was performed on a TA instruments machine (model Q500) in N_2_ flow. Optical microscopy images were obtained using a microscope by Zeiss (model Discovery V8) and a Leica (model DFC290). 90° Tape peel tests were performed with a 3M 396 Super Bond Film Tape (≈20 mm min^−1^).

The hysteresis measurements were performed on a Deben custom‐designed dual‐screw uniaxial testing machine. Samples were stretched till elongation ε_20_ = 20%, with the rate of 5 mm min^−1^, then deformation was released until tensile load was lower than 0.1 N. The residual elongation ε_r_ was recorded at each cycle (10 for each material). From the cyclic stress–strain curves, the recovered deformation was calculated as 1 −ε_r_/ε_20_.

## Conflict of Interest

The authors declare no conflict of interest.

## Supporting information

Supplementary

Supplementary

Supplementary

Supplementary
