# Supplementary material for: Carbon Nanofiber versus Graphene‐Based Stretchable Capacitive Touch Sensors for Artificial Electronic Skin
Source: Adv Sci (Weinh). 2017 Dec 27;5(2):1700587. doi: 10.1002/advs.201700587 (PMC5827098; doi:10.1002/advs.201700587)
Supplement: Supplementary file 1 — Supplementary [file ADVS-5-1700587-s002.pdf]

Copyright WILEY-VCH Verlag GmbH & Co. KGaA, 69469 Weinheim, Germany, 2017.

ADVANCED SCIENCE
Open Access

Supporting Information

for *Adv. Sci.,* DOI: 10.1002/advs.201700587

Carbon Nanofiber versus Graphene-Based Stretchable Capacitive Touch Sensors for Artificial Electronic Skin

Pietro Cataldi, Simeone Dussoni, Luca Ceseracciu, Marco Maggiali, Lorenzo Natale, Giorgio Metta, Athanassia Athanassiou, and Ilker S. Bayer**

Carbon Nanofiber vs Graphene-based Stretchable Capacitive Touch Sensors for Artificial Electronic Skin

Supporting Information

Pietro Cataldi[1*], Simeone Dussoni[2], Luca Ceseracciu[3], Marco Maggiali[2], Lorenzo Natale[2], Giorgio Metta[2], Athanassia Athanassiou[1], Ilker S. Bayer[1*]

[1]*Smart Materials, Istituto Italiano di Tecnologia, Via Morego 30, 16163 Genova, Italy*

[2]*ICub Facility, Istituto Italiano di Tecnologia, Via Morego 30, 16163 Genova, Italy*

[3]*Materials Characterization Facility, Istituto Italiano di Tecnologia, Via Morego 30, 16163 Genova, Italy*

Corresponding authors email address: pietro.cataldi@iit.it, ilker.bayer@iit.it

Raman Spectra Analysis of Carbon based Nanofillers

Figure S1 displays the Raman spectra of typical GnPs flakes and CnF nanowires. GnPs spectra present the characteristic signature of multilayer graphene material with mainly three peaks: the D located at 1360 cm^{-1}, the G at 1585 cm^{-1} and the 2D at 2700 cm^{-1}. The first and the last are activated with the presence of defects and are respectively the first and the second order relative to the breathing modes of sp^2 carbon bonds [1, 2]. In single layer graphene, the 2D peak is constituted of a single component, while it presents many in the form of graphene platelets or graphite[3]. The G peak is correspondent to the $E^2{}_g$ phonon at the Brillouin zone center[3, 4]. The thickness of the employed GnPs is estimated to be > of 9 layers in a previous work[5]. The CnF used in this works are treated with a graphitizing procedure (heat process at ≈3000°C) to generate a catalyst free product and maximize conductivity in composites. The CnF spectrum presents the same main peak of GnPs indicating its nanocrystalline structure compared

with amorphous carbon[(6)]. The ratio I(D)/I(G) is ≈ 0.1 and ≈0.6 respectively for GnPs and CnF, indicating a higher defect concentration for the nanowires.

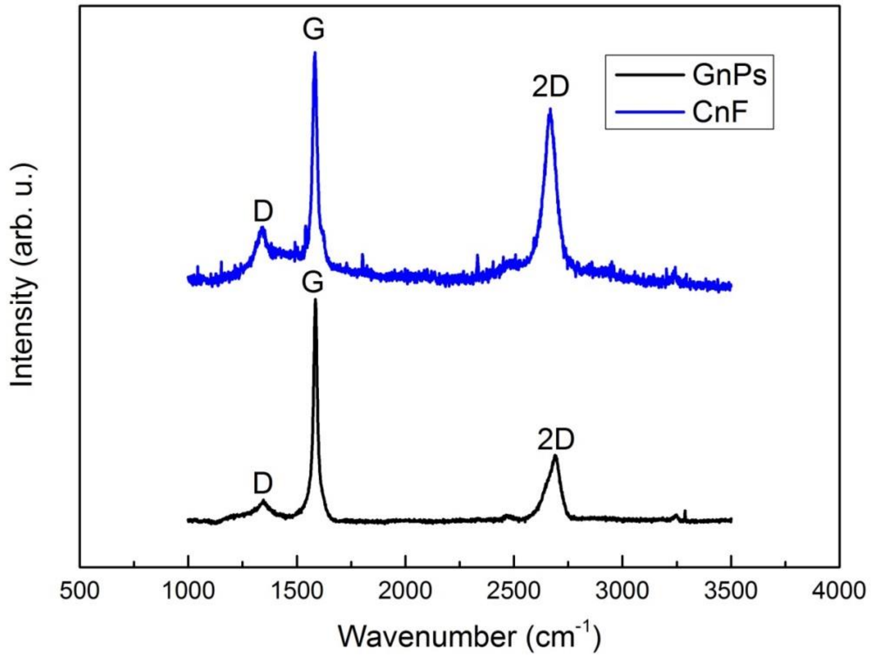

Figure S 1: Raman spectra of the carbon based filler. Both spectra are measured between 1000 and 3500 cm^{-1}.

Thermogravimetric Analysis

Figure S2 presents the Thermogravimetric Analysis (TGA) of the pure elastomer (Nitrile), of the polymer blend sprayed on the Nitrile (Polymer) and of two conductive nanocomposites containing 10 wt.% of GnPs or CnF. The degradation of a material is quantified considering the weight loss versus the temperature as shown in Fig. S2a. All the samples are thermally stable until ≈ 230°C. Considering such value, the heat gun temperature was set at ≈180-200°C, thus avoiding thermal degradation while

guaranteeing melting of the sprayed polymers. All the components (substrate and polymer blend matrix) degraded completely at ≈520 °C, preserving anyhow a residual percent mass around the 10% of its initial weight[7]. Interestingly, both the GnPs and CnF nanocomposites display a left-shift of the initial thermal degradation point. Such behavior is probably related to the nanofillers content which adsorb at the structural graphene/graphitic defects the –OH groups[8].

The first derivative of data in Fig. S2a is reported in Fig. S2b. Such graph highlights the degradation steps of the composites. Pure nitrile undergo a two steps degradation at ≈370-440 °C and at ≈440-530 °C[7]. The ink is composed by TPU (two degradation stages relative to the hard segment (between approx. 280 and 380 °C) and the soft segment (between approx. 380 and 440°C)[9]) and HIPS (single degradation step between 330 and 450 °C[10]). Therefore the composite without nanofillers (Polymer) presents a single degradation step which is the convolution of each degradation step presented above (Nitrile, TPU and HIPS). Introducing 10 wt.% GnPs or CnF, introduces peaks between 230 and 320 °C which are associated with the aforementioned defects of the graphitic material and the related –OH group.

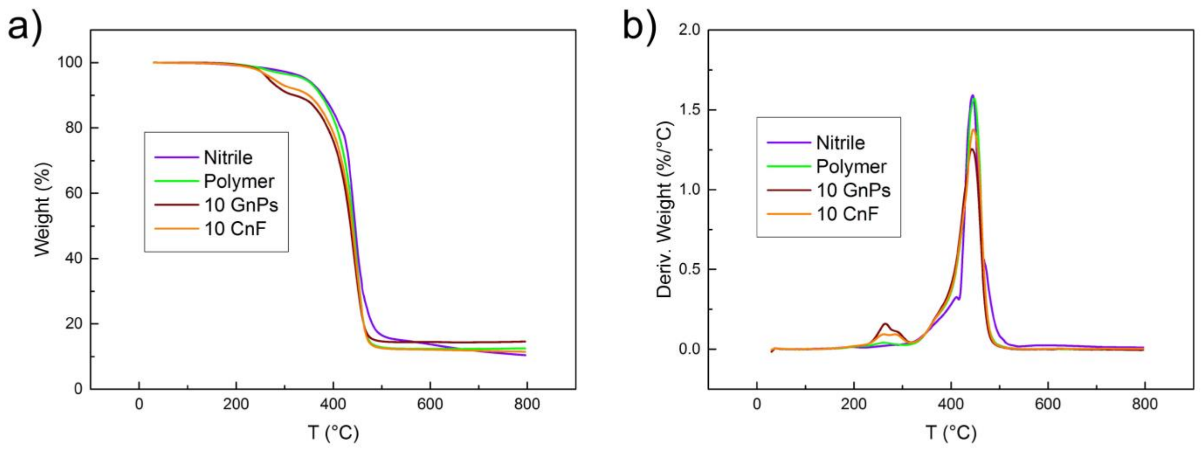

Figure S 2: Thermogravimetric analysis of the samples. a) Thermogravimetric weight loss measurements in nitrogen environment for various composites. b) The first derivative of the weight loss curve with respect to temperature.

Higher Magnification SEM images of the coatings Morphologies and Cross Sections

In Figure S3 we present SEM images of the details of the coating presented in the main text. Figure S3a and S3c display respectively the morphology and the cross section of the coating realized with 30 wt.% GnPs. Flakes of micrometric size are distinguishable in both the images (see black ellipse) and are distributed homogeneously through the sample. Figure S3b and S3d show respectively the morphology and the cross section of the coating fabricated with 30 wt.% CnF. Nanowires are distributed homogeneously at the top and through the thickness of the sample.

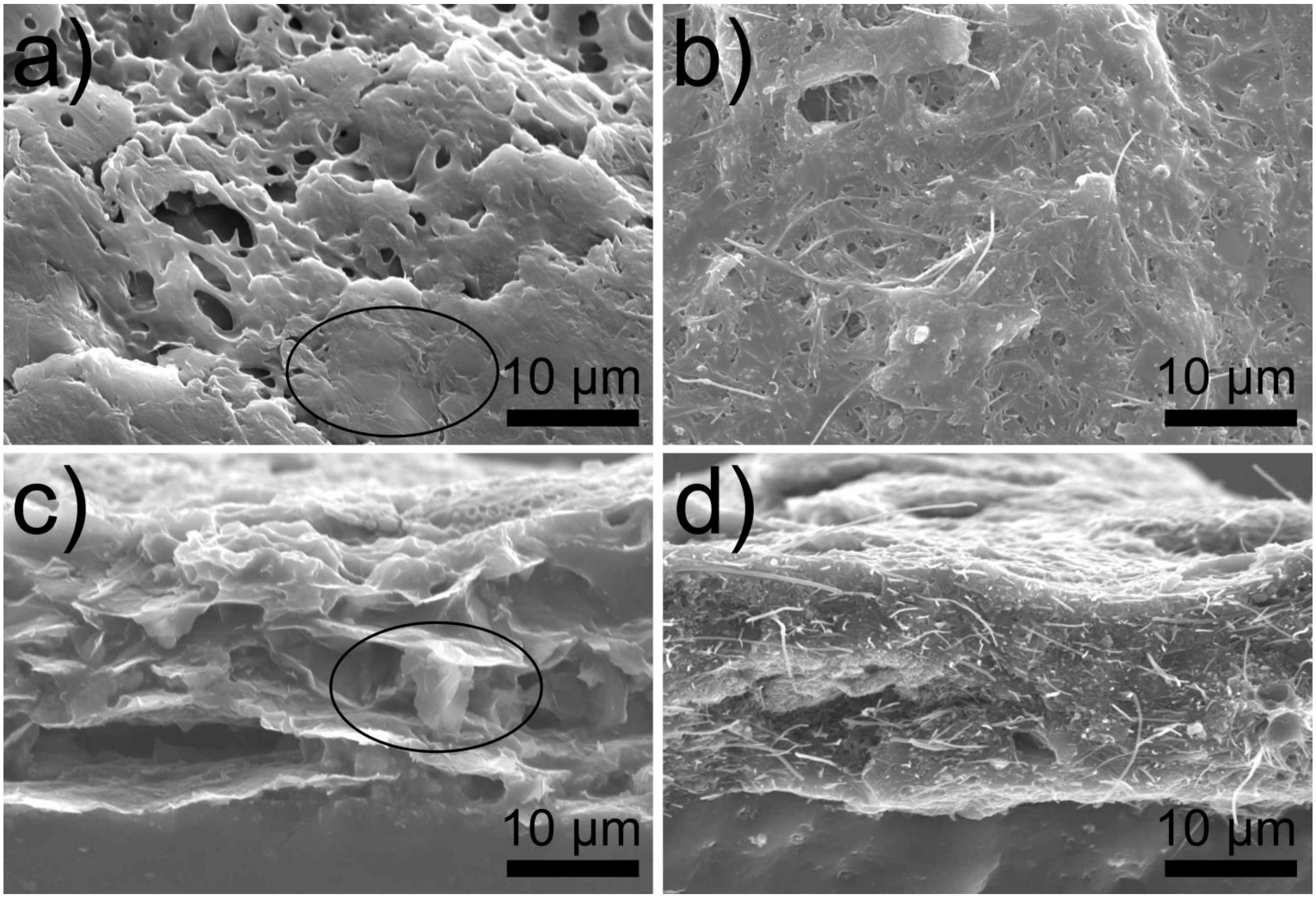

Figure S 3: SEM images of the morphology and cross section respectively of the GnPs (a,c) and CnF (b,d) based coatings.

Fourier Transformed Infrared Spectroscopy

We investigated the Fourier Transformed Infrared Spectroscopy (FTIR) of the substrate (Nitrile) and of the HIPS, TPU and polymer blend sprayed on the nitrile (respectively H-Nitrile, T-Nitrile and Polymer). The results are displayed in Figure S4. The bare Nitrile shows the typical peaks of the rubber family: in particular the C≡N stretching vibrations of acrylonitrile at 2230 cm^{-1} and the band at 960 cm^{-1} relative to C-H wagging vibration of butadiene (11). H-Nitrile exhibits the characteristic signature of polystyrene based materials, such as the aromatic ring bending between 700-800 cm^{-1}, C–H bending at ≈1440 and 1490 cm^{-1} and C–H stretching at ≈2840 and 2920 cm^{-1}(12). T-Nitrile sample presents the peaks of TPU at ≈1720 cm^{-1} (hydrogen bonded carbonyl) and at ≈1733 cm^{-1}(relative to carbonyl)(13). Also the peaks at 2850 and 2930 cm^{-1} (respectively the symmetric and asymmetric vibration of the C-H_2 group) and at 3330 cm^{-1} (N-H group in urethane) are visible(14). For both H-Nitrile and T-Nitrile samples, nitrile rubber chemical signature was not found. This is due to the thickness of the sprayed coatings (in the order of ten μm) which prevents penetration of the IR light to the elastomeric substrate. The spectrum of the Polymer composites shows all the major peaks relative to the single polymer employed for the blend (HIPS and TPU) without important shifts. Again, the nitrile signal is absent due to the thickness of the sprayed coating. Adding the nanofillers introduces more noise and a decrease in the relative peaks intensity. Since no significant modification are observed, there appear to be no chemical interaction between GnPs or CnFs and the polymers in the nanocomposites.

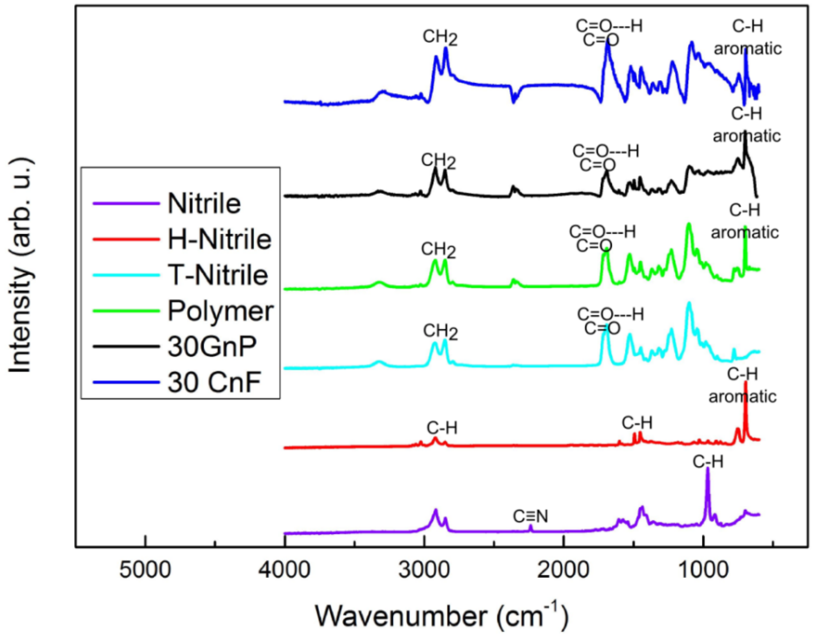

Figure S 4: FTIR of the bare nitrile (purple), of the HIPS (red), TPU(cyan), and their polymer blend (green) sprayed on the elastomer and of the nanocomposites with 30wt.% either of GnPs (blue) or CnF (black).

Peel Tests for coating adhesion

Tape peel tests are accomplished to validate the adhesion strength of a coating on a substrate. We have applied tape peel adhesion tests to pure TPU-HIPS polymers, CnFs- and GnPs- based nanocomposite having the best electrical conductivity (30 wt.% filler concentration). In Figure S5 we display a typical peeling distance vs force graph obtained for the CnF-based sample. The adhesion force (resulting to be >1.0 N/m for all the tests performed) was calculated performing a linear fit on the flat region of the figure. Tests performed with GnPs or with the bare polymer coating were producing similar results.

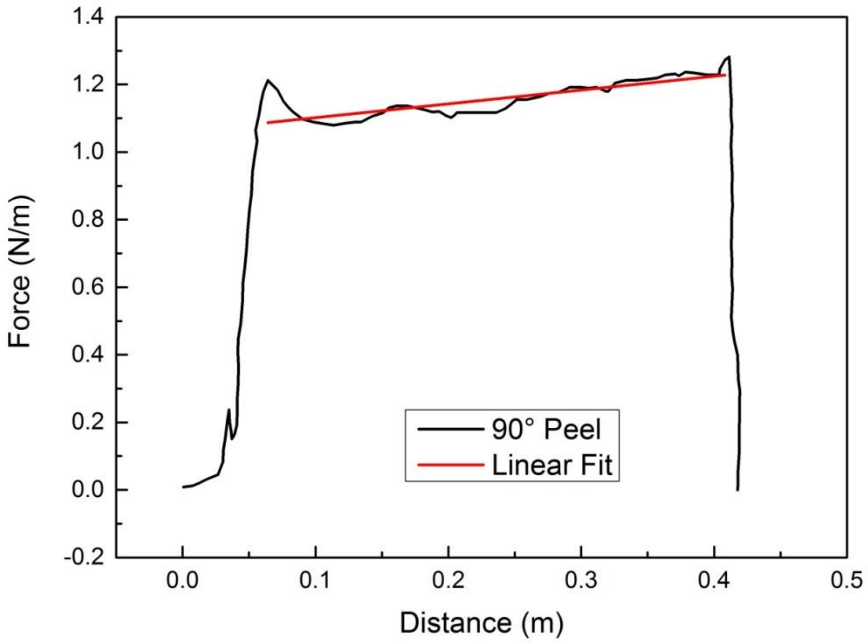

Figure S 5: Typical 90° peeling tests conducted on CnF-based nanocomposites.

Current-Voltage Curves

In Figure S6 we show typical current-voltage (I-V) trends obtained with our nanocomposites below and above electrical percolation. Below this threshold, the I-V curves display an highly hysteretic behavior characteristic of electrical insulator. We report only exemplificative results relative to the 0.5 wt.% CnF and 1wt.% GnPs based nanocomposite. After percolation a typical ohmic I-V response is reached. This time we showed as an example the 10wt.% CnF and GnPs loaded samples.

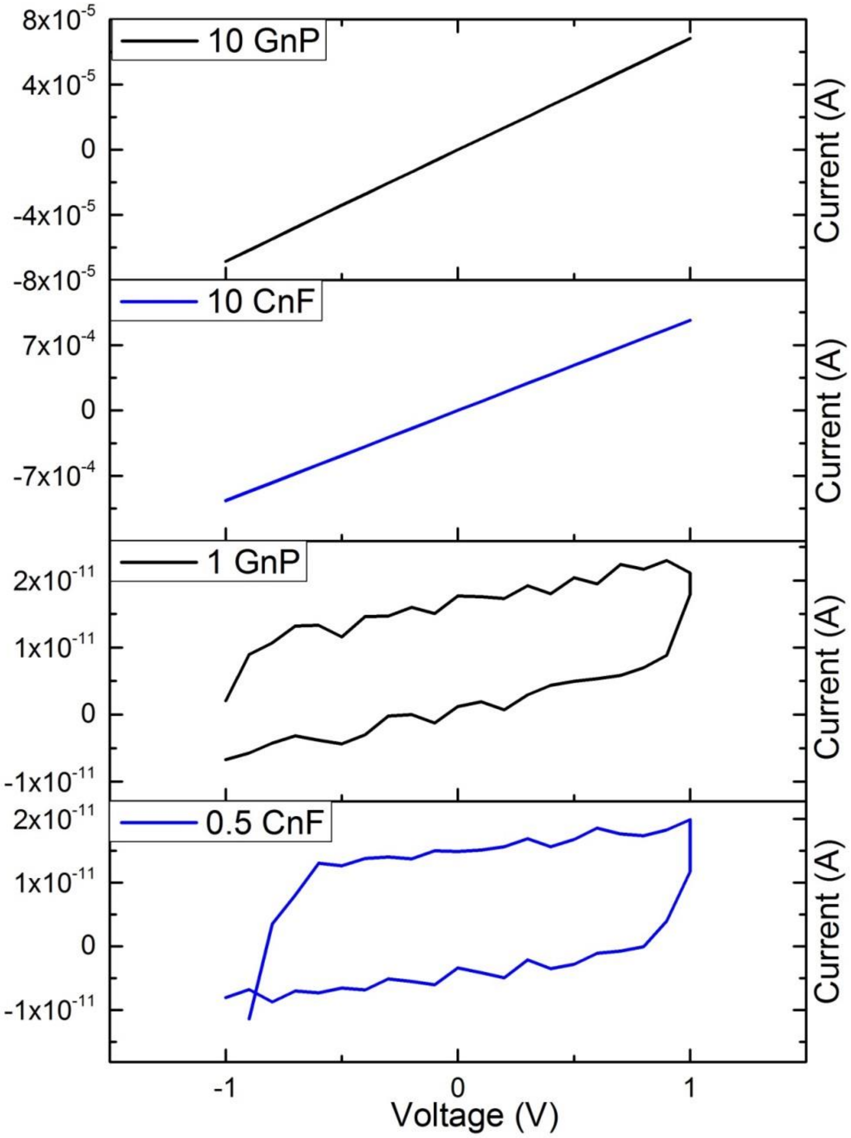

Figure S 6: I-V curves of the nanocomposites before and after percolation.

SEM images under stretching for 30 wt.% GnPs based Nanocomposites

Figure S7 shows details on the cracking phenomenon observed on the GnPs based nanocomposites. Increasing the amount of GnPs leads to an earlier cracking behavior with elongation. We show details of the 30 wt.% GnPs loaded sample. At 15% elongation no cracks were present on the sample.

Afterwards large cracks were starting and at 60 % elongation the cracks are numerous on the nanocomposite surface.

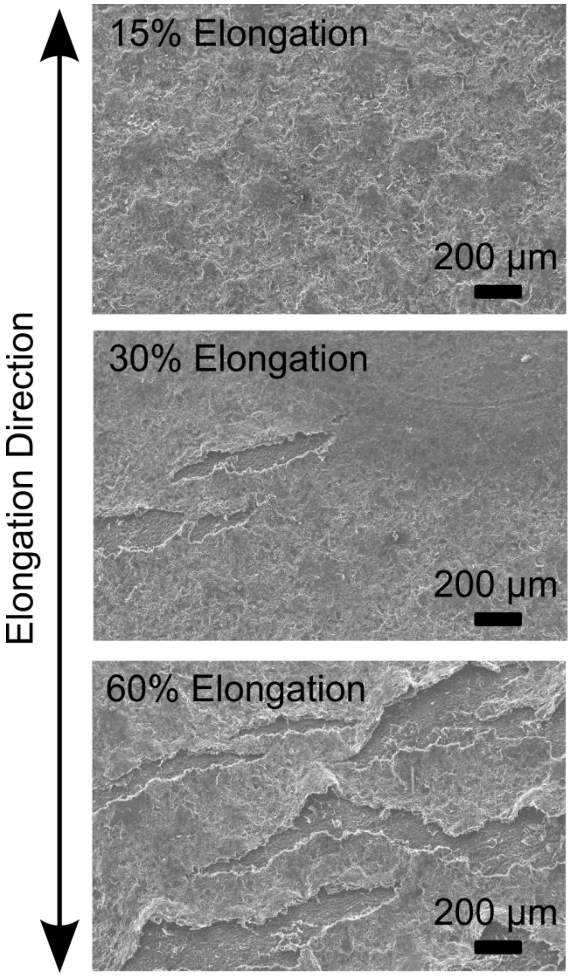

Figure S 7: SEM images of the cracking mechanism of the 30 wt.% GnPs loaded sample with increasing elongation.

Hysteresis Measurements

We evaluated the reversibility of deformation of our artificial skin materials through cyclic mechanical tests. All tests were performed on a Deben custom-designed dual-screw uniaxial testing machine. Samples were stretched with the rate of 5 mm/min, until elongation $\varepsilon_{20} = 20\%$, then deformation was

released until tensile load was lower than 0.1 N, and the residual elongation ε_r was recorded. Each test had a duration of 10 cycles. From the cyclic stress-strain curves, the recovered deformation was calculated as $1 - \varepsilon_r/\varepsilon_{20}$.

The stiffening effect due to either nanofiller is visible in the cyclic stress-strain curves (Fig. S8a), as well as the lower deformation recovery of the CnF 30 sample, compared to PMix and 30 GnP materials. The trend as a function of the number of cycles show also a faster decaying of the recovered deformation, with values, after 10 cycles, about 5 times lower than the unfilled PMix and the 30 GnP materials (Fig. S8b)

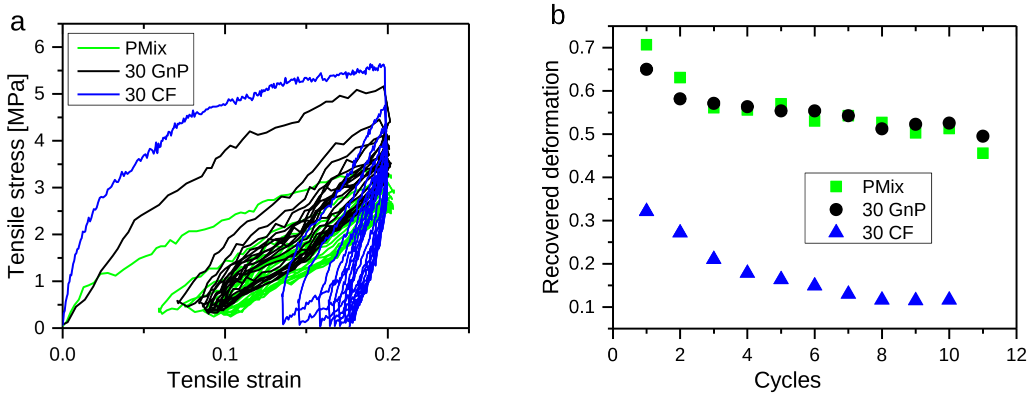

Figure S8: Oligo-cyclic characterization of artificial skins: a) cyclic stress-strain curves of selected materials, showing the lower recoverable deformation of CnF 30; b) Relative recovered deformation as a function of the number of cycles.

SEM Images of the GnPs based samples released back to rest position (0)

In Figure S9 we show a sample loaded with 30 wt.% GnPs at rest length after 100% elongation.

The cracks formed during elongation are contacted back.

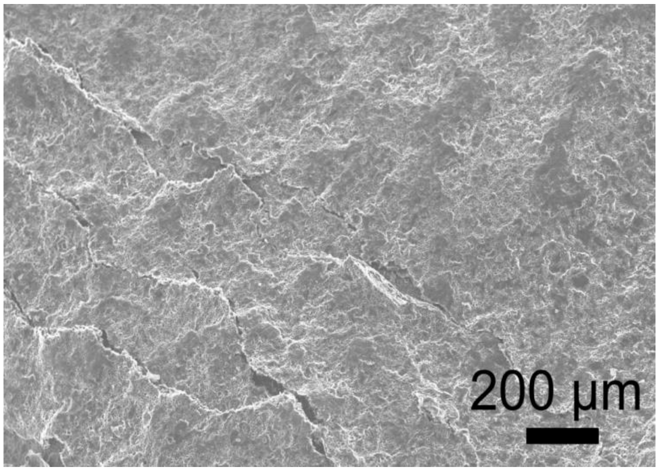

Figure S 9: Sem image of the 30 wt.% GnPs loaded sample after relaxation. The sample was previously elongated at 100% elongation.

Optical Microscopy of Laser Micromachined Holes before and after Spray

In figure S10 are displayed optical microscopy images of a square shaped hole (approximately 300 µm side) micromachined on the pure nitrile substrate. The hole is shown before (Fig. S10a) and after (Fig. S10b) spray coating of a 30 wt.% GnPs based conductive ink. After spray the hole (via) is filled with the conductive ink, rendering the inside conductive. If spray coating was performed on both sides, electrical contact was established between the top and bottom part of the pure nitrile. Equivalent results were obtained with CnF based conductive inks.

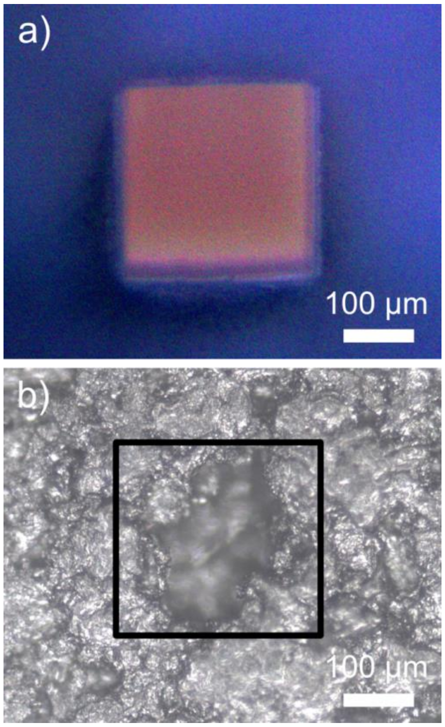

Figure S 10: Optical microscopy images of a hole created with micromachining technique before and after spray coating of a 30 wt.% GnPs based dispersion.

Bottom of the Stretchable Tactile Sensor

In Figure S11 is presented the bottom design of the sprayed tactile device. Such design can be obtained with both CnF and GnPs based conductive inks. We show a GnPs based coating 30 wt.% loaded.

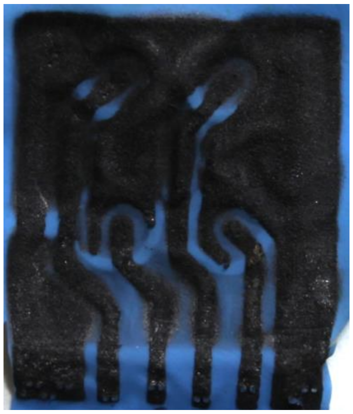

Figure S 11: Bottom of the tactile sensor. The four lines bring the signal to electronics.

GnPs-based Stretchable Tactile Sensor under Stretch

In Figure S12 is shown the GnPs-based stretchable tactile sensor under stretch. Cracks appear when stretched.

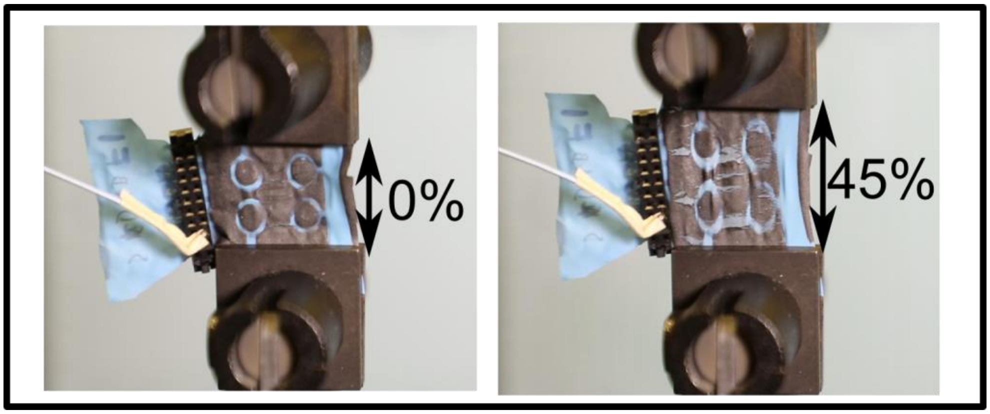

Figure S 12: Device GnPs-based at zero elongation and 45 % stretch.

References:

(1) A. C. Ferrari, D. M. Basko, *Nat. Nanotech.* **2013**, *8*, 235.

(2) A. C. Ferrari, J. Robertson, *Phys. Rev. B* **2001**, *64*, 075414.

(3) A. C. Ferrari, J. C. Meyer, V. Scardaci, C. Casiraghi, M. Lazzeri, F. Mauri, S. Piscanec, D. Jiang, K. S. Novoselov, S. Roth, A. K. Geim, *Phys. Rev. Let.* **2006**, *97*, 187401.

(4) A. C. Ferrari, J. F. Robertson, *Phys. Rev. B* **2000**, *61*, 14095.

(5) P. Cataldi, I. S. Bayer, G. Nanni, A. Athanassiou, F. Bonaccorso, V. Pellegrini, A. E. del Rio Castillo, F. Ricciardella, S. Artyukhin, M.-A. Tronche, Y. Gogotsi, R. Cingolani, *Carbon* **2016**, 331.

(6) A. C. Ferrari, *Solid State Comm.* **2007**, *143*, 47.

(7) M.A. Kader, K. Kim, Y.- S. Lee, C. Nah, *J. Mater. Sci.* **2006**, *41*, 7341.

(8) C. –C. Teng, C.- C. M. Ma, C.- H. Lu, S.- Y. Yang, S.- H. Lee, M.- C. Hsiao, M.- Y. Yen, K.- C. Chiou, T.- M. Lee, *Carbon* **2011**, *49*, 5107.

(9) M. A. Hood, B. Wang, J. M. Sands, J. J. La Scala, F. L. Beyer, C. Y. Li, *Polymer* **2010**, *51* 2191.

(10) E. H. Agung, S. M. Sapuan, M. M. Hamdan, H. M. D. K. Zaman, U. Mustofa, *Int. J. Phys. Sci.* **2011**, *6* 2100.

(11) S. Gunasekaran, R. K. Natarajan, A. Kala, *Spectrochimica Acta Part A: Molecular and Biomolecular Spectroscopy* **2007**, *68*, 323, 2007.

(12) Y. Lin, K. M. Ng, C. –M. Chan, G. Sun, J. Wu, *J. Col. Interface. Sci.* **2011**, *358*, 423.

(13) A. Pattanayak, S. C. Jana, *Polymer* **2005**, *46*, 5183–5193.

(14) H. –Y. Mi, M. R. Salick, X. Jing, B. R. Jacques, W. C. Crone, X. –F. Peng, L.- S. Turng, *Mater. Sci. Eng.: C* **2013**, *33*, 4767.
